# High electrochemical performance of nickel cobaltite@biomass carbon composite (NiCoO@BC) derived from the bark of *Anacardium occidentale* for supercapacitor application†

**DOI:** 10.1039/d3ra08138a

**Published:** 2024-02-14

**Authors:** Modou Diop, Baye Modou Ndiaye, Sokhna Dieng, Balla D. Ngom, Mohamed Chaker

**Affiliations:** a Institut National de la Recherche Scientifique (INRS), Centre – Énergie Matériaux Télécommunications (EMT) 1650, Boul. Lionel Boulet, Varennes Québec J3X 1P7 Canada mohamed.chaker@inrs.ca; b Laboratoire de Photonique Quantique, d'Énergie et de Nano-Fabrication (LPQEN), Faculté des Sciences et Techniques, Université Cheikh Anta Diop de Dakar (UCAD) B.P. 5005 Dakar-Fann Dakar Senegal bdngom@gmail.com

## Abstract

Biomass carbon-based materials are highly promising for supercapacitor (SC) electrodes due to their availability, environment-friendliness, and low cost. Herein, an easy energy-saving hydrothermal process was used to produce NiCo_2_O_4_/NiOOH (NiCoO) composites with biomass carbon (BC) derived from the bark of *Anacardium occidentale* (AO) at different synthesis time durations (2 h, 4 h, 8 h, 16 h). The structural and morphological properties of the samples were analysed using XRD, Raman spectroscopy, XPS, SEM, TEM and BET, and the results exhibit the presence of carbon inserted into the nickel–cobalt hydroxide matrix. The NiCoO@BC composite synthesized in 4 h (NiCoO@BC(4 h)) displays a good specific capacitance of 475 F g^−1^ at 0.5 A g^−1^ and a low equivalent series resistance (ESR) value of 0.36 Ω. It shows a good coulombic efficiency of 98% and retains 86% of the capacitance after 4000 cycles. The asymmetric supercapacitor (ASC) device (NiCoO@BC(4 h)//AC) assembled using activated carbon (AC) as a negative electrode displays 20 W h kg^−1^ energy density and 900 W kg^−1^ power density at 1 A g^−1^. The stability test shows a good coulombic efficiency of 99% and 78% capacitance retention after 15 000 cycles. These findings imply that NiCoO@BC composites have outstanding electrochemical properties, making them suitable as SC electrode materials.

## Abstract 2

Nickel cobaltite hydroxide NiCo_2_O_4_/NiOOH (NiCoO) with biomass carbon (BC) are successfully synthesized using the bark of *Anacardium occidentale* extract as a carbon source *via* an energy-saving hydrothermal process. The carbon from biomass greatly enhances the electrochemical performances of NiCoO.

## Graphical abstract

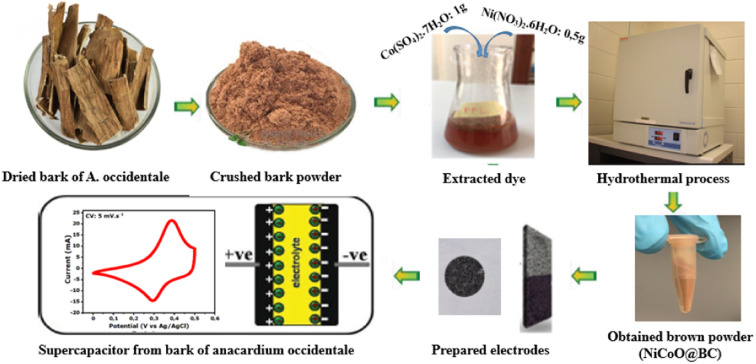

## 1. Introduction

The current energy crisis and the reliance on fossil fuels for production highlight the need for a paradigm shift towards eco-friendly and renewable energy sources. Sunlight and wind are great alternative options.^1^ However, these intermittent sources require electrochemical energy storage devices to circumvent discontinuity.^2^ Supercapacitors (SCs) are currently considered attractive energy storage devices due to their good stability, low maintenance cost, high power density, and eco-friendliness compared to battery devices.^3–6^ SCs may be divided based on the mechanism of energy storage into two main groups:^7^ electric double-layer capacitors (EDLCs) store electrical energy through ion adsorption^8^ and faradaic capacitors or pseudo-capacitors store electrical energy through fast redox reactions.^9,10^ EDLCs work primarily with carbon-based materials characterized by high surface area, mechanical resistance, and good electrical conductivity, which result in long life cycle and high power density.^11,12^ However, their commercial application is questioned by the relatively low specific capacitance.^13,14^ Metal oxides/hydroxides and conductive polymers are generally utilized for faradaic capacitor materials. Their ability to display various oxidation states with significant charge transfer reactions makes them capable of achieving high specific capacitance and energy density.^15^ However, despite their potential benefits, these materials have low stability and poor electrical conductivity, which are not useful in practical applications.^16,17^

According to previous studies, ruthenium oxide (RuO_2_) is amongst the earliest metal oxides to be studied as a traditional pseudocapacitive electrode material.^18^ However, ruthenium in its elemental form is costly, hindering the achievement of commercial applications with high-efficiency rates.^19^ Therefore, some inexpensive transition metal oxides, including NiO,^20^ SnO_2_,^21^ MnO_2_,^22^ and Co_3_O_4_,^23^ were manufactured as alternative materials. Among these metal oxides, Co_3_O_4_, with a theoretical specific capacitance of 3560 F g^−1^, is a fascinating electrode material for SCs.^24^ Nevertheless, the cycling stability of Co_3_O_4_ is still a challenge for practical applications. The spinel cobaltite MCo_2_O_4_ (M = Ni, Cu, Mn) derived from Co_3_O_4_ is considered promising due to the mixed valence metal cations, which provide better electrochemical performances compared to single-metal oxides.^25,26^ Mesoporous Co_3_O_4_ nanocubes reported by Liu *et al.*^23^ exhibit 220 F g^−1^ capacitance and good stability. Flower-like MnCo_2_O_4_ hollow microspheres^27^ and pod-like MnCo_2_O_4.5_ microstructures^19^ synthesized using a solvothermal method yield 235.7 and 321 F g^−1^ of capacitances and 93% (after 2000 cycles) and 87% (after 4000 cycles) capacitance retention, respectively. Porous hexagonal NiCo_2_O_4_ nanoplates synthesized by Pu *et al.*^28^ exhibit a capacitance of 294 F g^−1^ and 89.8% capacity retention after 3000 cycles.

Studies have also reported that porous carbon combined with metal oxides increases the electrochemical activity and stability of the obtained composite. Recently, various carbon-based materials have been utilized to enhance the properties of metal oxides. The most used are reduced graphene oxide (rGO),^29–31^ graphene,^32–34^ activated carbon,^35–37^ g-C_3_N_4_,^38,39^ and carbon nanotubes (CNT).^40^ They have excellent properties, such as large surface area and good conductivity, but their fabrication process from commercial precursors is however costly and environmentally harmful. Actually, biomass is a compelling choice for creating carbon-based materials due to its renewability, sustainability, environmental friendliness, and low cost.^41,42^

A variety of biomass, including orange peel,^43^ watermelon,^44^ peanut shell waste,^45^ flower,^17^ dead leaves,^46^ rice husks,^47^ and *Hibiscus sabdariffa*,^16^ have been used to synthesize metal oxides/carbon composites for supercapacitor applications.^48–50^ For example, Qian Li *et al.*^17^ fabricated Ni(OH)_2_ on carbon microtubes from willow catkins. The asymmetric device in 6 M KOH displayed energy and power densities of 37 W h kg^−1^ and 750 W kg^−1^, respectively. Shan Liu *et al.*^51^ produced lignin-derived porous carbon nanofibers (Co_3_O_4_–CNFs) *via* electrospinning and demonstrates a capacitance of 369 F g^−1^ at 0.1 A g^−1^. Nan *et al.*^52^ also obtained NiCo_2_O_4_ with carbon from poplar catkins. The as-prepared electrode exhibits a capacitance of 1538 F g^−1^ (922.9 C g^−1^) at 1 A g^−1^, which retains 91.4% of its value after 5000 cycles.

Additionally, biomass liquid extracts can also be employed, besides carbon sources, as green catalysts to synthesize metal oxide nanoparticles. Kundu *et al.*^53^ used *Hydrangea paniculata* flower extracts to fabricate nickel oxide nanoparticles (NiO-NPs) for supercapacitor electrode materials. For the same purpose, B. D. Ngom *et al.*^16^ also successfully synthesized vanadium pentoxide carbon composite (V_2_O_5_@C) derived from the natural *Hibiscus sabdariffa* (HS) family using a green solvothermal process. They concluded that a high amount of carbon from the HS extract was incorporated into the V_2_O_5_ matrix, which improved the electrochemical performances.

The synthesis procedures of these carbon-based materials are mostly time-consuming and energy-intensive, and therefore, are very expensive for large-scale applications. In this regard, the extract from plant waste can be used as a renewable carbon precursor and integrated into a nickel–cobalt matrix for low-cost and sustainable electrode material.

Therefore, *Anacardium occidentale* (AO), a cultivable plant belonging to the family of Anacardiaceae as natural and renewable biomass, has been used sparingly to fabricate carbon-based materials. This tropical plant can grow up to 15 m tall. It has a twisted and thick trunk with woody branches.^54^ Several studies performed on leaf, stem, and bark extracts to isolate chemical compounds showed that the different parts contain many bioactive molecules, some of them being represented in Fig. S1.†^54–56^

In the present study, we prepared a composite nickel cobaltite hydroxide, NiCo_2_O_4_/NiOOH (NiCoO), with biomass carbon (BC) from the bark of *Anacardium occidentale* (AO) *via* a facile shot-step hydrothermal process at low temperature (150 °C) for different synthesis time durations of 2 h, 4 h, 8 h, 16 h. The dye extracted from AO containing bioactive molecules like phenolic acid functional groups was employed as a green catalyst for metal oxide fabrication^16,57^ and carbon source. The filtered waste of the bark is further used to fabricate activated carbon and extract lignin and cellulose. In this regard, we will value all the parts of the bark to produce sustainable and renewable materials for supercapacitor electrodes. We also consider the importance of the survival of the plant and the large-scale application. During the collection, we do not remove the bark totally, so the plant does not suffer from any problems and develops new bark in a short period. Actually, the quantity used at a laboratory scale is not so important; however, the AO is a cultivable plant that can be easily developed for large-scale applications.

The BC incorporated into nickel–cobalt hydroxide was examined using XPS, Raman, and EDS mapping images.

The electrochemical performance of the NiCoO@BC composite analyzed firstly with a three-electrode system revealed better specific capacitance and stability than pristine NiCoO without biomass carbon. Moreover, the NiCoO@BC(4 h) composite as a positive electrode in an asymmetric supercapacitor NiCoO@BC(4 h)//AC with AC in the opposite electrode provides good energy/power density. This work offers fresh ideas for future advances in developing electrode materials for supercapacitors using energy-saving processes and promotes biomass use for energy storage.

## 2. Material synthesis

### 2.1. Materials

Reagents Co(SO_4_)·7H_2_O (purity 99%), Ni(NO_3_)_2_·6H_2_O, (purity 99%), and potassium hydroxide (KOH, min 85%) purchased from Sigma-Aldrich were directly used without any purification. The barks of *Anacardium occidentale* (AO) were collected in Senegal. A 3D scaffold nickel foam (thickness: 1.6 mm, areal density: 420 g m^−2^) was used as material support and current collector.

### 2.2. Preparation of the NiCoO@BC composite

**Fig. 1:**
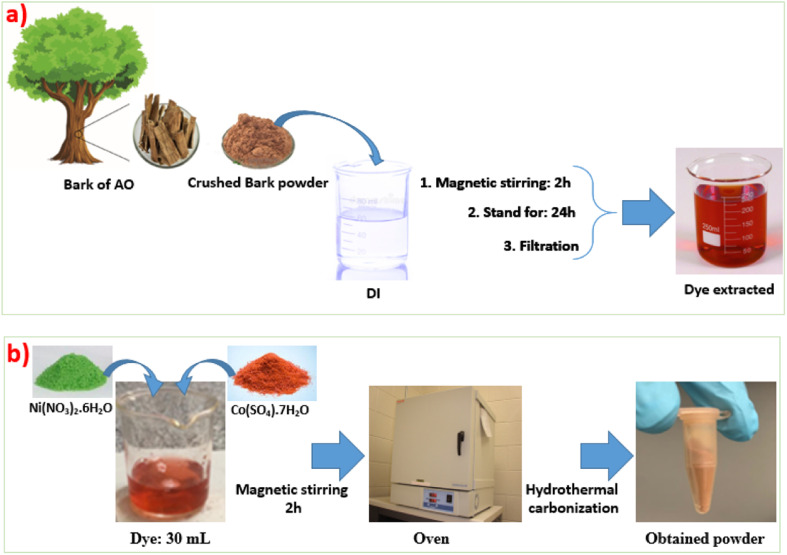
(a) Schematic process for the extraction of dye from *Anacardium occidentale* (AO), (b) hydrothermal synthesis steps of NiCoO@BC.

Two steps were used to synthesize NiCoO@BC composites from the barks of AO: dye extraction and hydrothermal synthesis. The barks were first air-dried naturally, crushed into a fine powder using a blender, and 3 g of the crushed barks were mixed into 150 mL of distilled water. The mixture, after stirring for 2 h, was left to stand for 24 h at ambient temperature to make sure that bioactive compounds were fully extracted and then filtered to obtain the dye, as shown in Fig. 1a. Then, hydrothermal synthesis was performed using 1 g of Co(SO_4_)·7H_2_O, 0.5 g of Ni(NO_3_)_2_·6H_2_O, and 30 mL of the above dye obtained from AO. This mixture was dissolved under continuous magnetic stirring for up to 2 hours to homogenize the solution and then transferred into an electric oven at 150 °C for different synthesis time durations to obtain a brown color powder as shown in Fig. 1b and named nickel cobaltite oxide biomass carbon (NiCoO@BC). The same steps were used to fabricate either pristine NiCoO with no added dye as a sample reference or other NiCoO@BC samples at different synthesis time durations of 2 h, 4 h, 8 h, and 16 h.

### 2.3. Synthesis of activated carbon (AC)

A raw material, peanut shell waste (PSW), was used to fabricate AC through a conventional direct pyrolysis technique reported by Sylla *et al.*^45^ Briefly, the PSW was collected, washed with deionized water, dried under direct sunlight for over 12 h, and crushed into fine powder. The mixture of 5 g of PSW powder, 1 g of NaCl, and 0.5 g of urea with deionized water (a few drops) was dried at 80 °C overnight and then transferred into a furnace under a 250 sccm argon flow for 2 h at 600 °C. The obtained black product was mixed with porosity-enhancing KOH agents and submitted to pyrolysis at 850 °C for 1 h under 250 sccm argon flow. After filtering, the solid material was washed with deionized water until neutral pH and then dried at 80 °C for 12 hours.

## 3. Characterization methods

Structural and morphological properties were examined by (i) X-ray diffraction measurements (Bruker D8 Advance, *λ* = 1.542 Å), (ii) Raman spectroscopy (laser wavelength *λ* = 473.1 nm), (iii) X-ray photoelectronic spectrometry (model: VG Escalab 220i XL, Al Kα 1,2 polychromatic source *hν* = 1486.6 eV), (iv) scanning electron microscopy (Tescan Vega3 LMH) and (v) transmission electron microscopy (model: JEM 2100 F, 200 kV). The porosity was evaluated by nitrogen adsorption–desorption measurements (relative pressure *P*/*P*_0_: from 0.0 to 1.0) using the Brunauer–Emmett–Teller analysis (BET: Quantachrome), and the specific surface area was determined by BET theory. The electrochemical study was performed using a Biologic VMP 300 instrument, reference electrode (Ag/AgCl: 3 M KCl saturated), and platinum plate counter electrode.

## 4. Preparation of electrode material

To prepare the working electrode, conductive carbon black (10%), active material (80%), and polyvinylidene fluoride (10%) as a binder were mixed with a low-viscosity solvent NMP (*N*-methyl-2-pyrrolidone) to disperse the active material.^58^ The obtained slurry was pasted on a current collector (nickel foam) and then dried for 12 h at 60 °C in an oven to obtain the electrode. The active material was coated on a surface of about 1 cm^2^ for all the electrodes. Then, a similar process was employed to fabricate positive (NiCoO@BC(4 h)) and negative (AC) electrodes for assembling a full device (Swagelok system; stainless steel plungers) using filter paper as electrode separator in 6 M KOH.

## 5. Results and discussion

### 5.1. Material characterizations

**Fig. 2:**
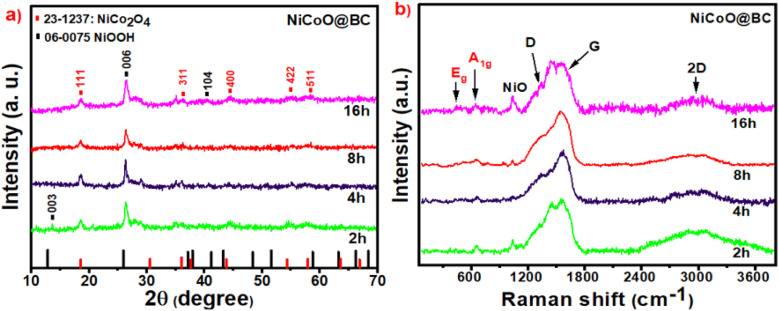
(a) XRD peak patterns and (b) Raman spectra of NiCoO@BC composites at different synthesis time durations of 2 h, 4 h, 8 h, and 16 h.

XRD spectra of the pristine and the composites prepared in 2 h, 4 h, 8 h, and 16 h synthesis time durations, as shown in Fig. 2a and S2a,† are in agreement with the hexagonal structure of NiOOH and the spinel NiCo_2_O_4_ peak patterns. The prominent peaks at 18.4°, 36.5°, 44.0°, 55.0°, and 59.0° were ascribed respectively to (111), (311), (400), (422), and (511) planes of spinel NiCo_2_O_4_ which belong to the *Fd*3̄*m* space group system, a cubic crystal structure with face-centered identified through JCPDS file (no. 20-0781).^59,60^ NiCo_2_O_4_ has a spinel structure with Co^2+^ cations located in tetrahedral sites, while Ni^3+^ and Co^3+^ cations are situated in octahedral sites.^59^ NiOOH exhibits three peaks at 12.0°, 25.2°, and 41.0°, which are consistent with the (003), (006), and (104) planes of the hexagonal structure according to Database (ICSD) Card No. 06-0075.^61–63^

The diffraction peaks of the obtained carbon are not observed because of the amorphous nature of the biomass carbon. Nevertheless, the presence of carbon is confirmed through Raman spectra, as shown in Fig. 2b, which shows three bands: G, D, and 2D. The D and G bands are observed at 1320 and 1560 cm^−1^, respectively, in two broad overlapping peaks.^14^ The G band correlated with the in-plane stretching sp^2^ carbon vibration in graphite-like structure is beneficial for charge storage.^64^ As for the D band, it refers to disordered sp^2^ carbon, which is valuable for electrical conductivity.^64^ Furthermore, in the range 2600–3000 cm^−1^, a broad band appears as the Raman signature of the carbon 2D-band (an overtone of the D band), confirming the presence of amorphous carbon in the composites.^65^ Fig. S2b† compares the Raman spectra of pristine NiCoO without biomass carbon and the NiCoO@BC(4 h) composite. As displayed, the presence of carbon peaks in the composite confirms the incorporation of carbon from the bark of AO extract into the nickel–cobalt hydroxide matrix. The intensity ratio (*I*_D_/*I*_G_) for the sample NiCoO@BC(4 h) was calculated to be 0.82, implying that numerous defects exist in the carbon amorphous structure. According to Nan *et al.*,^52^ multiple structural defects can lead to improved conductivity by creating more attachment sites for metal ions. Previous studies^66,67^ have demonstrated A_1g_, E_g_, and three F_2g_ bands as the five Raman active modes in the ideal spinel structure. The octahedral and the tetrahedral cation vibration modes F_2g_, E_g_, and A_1g_ (for CoO and NiO) are observed at 245, 486, and 630 cm^−1^, respectively.^28^ The other band at 3400 cm^−1^ in the pristine material refers to the OH vibration mode from NiOOH.^68^

**Fig. 3:**
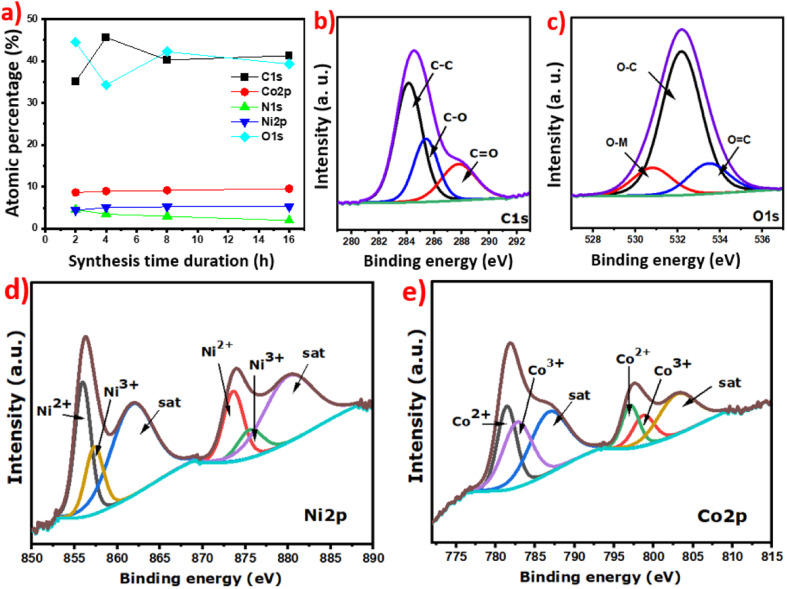
(a) Atomic percentage *versus* synthesis time duration (2 h, 4 h, 8 h, 16 h) and (b)–(e) fitted spectra of C, O, Co, and Ni elements of NiCoO@BC(4 h).

The XPS measurement was performed to identify the elemental composition of the NiCoO@BC composite and to confirm the presence of carbon. The survey spectra shown in Fig. S3a† exhibit the peaks of O, C, Co, and Ni elements. These results are consistent with those obtained through XRD and Raman patterns. Therefore, in order to investigate the carbon amount in the different samples, the atomic percentage is calculated from XPS spectra and represented *versus* the synthesis time durations in Fig. 3a. The NiCoO@BC(4 h) sample heat-treated at 4 h contains higher carbon (45%) and lower oxygen concentrations than the other samples.

The percentage of carbon increases from 2 h to 4 h synthesis duration and decreases beyond this time limit. This result reveals that a 4 h synthesis duration at 150 °C is the optimized reaction condition required to maximize the amount of carbon. The decrease in the amount of carbon is due to the formation of CO_2_, which is released in gaseous form after the optimum synthesis time duration.

Fig. 3b–e displays the XPS deconvolution of C 1s, O 1s, Ni 2p, and Co 2p for the NiCoO@BC(4 h) composite. The core-level spectra of C 1s and O 1s peaks show the binding energies C–O, C–C, and C=O in the carbon matrix and O-metal bonds (Fig. 3b and c).

The high-resolution spectrum of Ni 2p is fitted into two peaks at 856 eV and 858 eV corresponding to the Ni^2+^ and Ni^3+^ oxidation states, respectively, and a less intense satellite peak appears at 862 eV (Fig. 3d).^34^ The Co 2p spectrum fitted in a similar way shows two oxidation states, Co^2+^ at 781 eV and Co^3+^ at 783 eV, and a satellite peak at 787 eV (Fig. 3e). These outcomes mean that Co^3+^/Co^2+^ and Ni^3+^/Ni^2+^ redox couples coexist in the metal oxide structure of the NiCoO@BC composites. These redox couples create multiple electroactive sites, enhancing the electrochemical performance.^38,52^ Small amount of nitrogen noticed in the composites is originated from the bark of AO, which has 2.1% nitrogen content (Fig. S3b†). That percentage of nitrogen and carbon in the bark of AO is more than those in other biomass parts reported by Raimie H. H. Ibrahim *et al.*,^69^ as shown in Table S1.† These nitrogen atoms can supply electron donors, which improve the wettability and the electrical conductivity of the NiCoO@BC composite as electrode material.^70^ Additionally, the nitrogen introduced into the carbon matrix has the potential to generate significant pseudocapacitance through faradaic redox reactions in aqueous electrolytes.^14,71^ The high-resolution spectra of all the elements in the composites for different synthesis time durations are presented in Fig. S4.† As observed, the nitrogen peak decreases significantly when the synthesis time duration is increased and almost disappears at 16 h, corresponding to the appearance of metal–carbon binding (M–C).

**Fig. 4:**
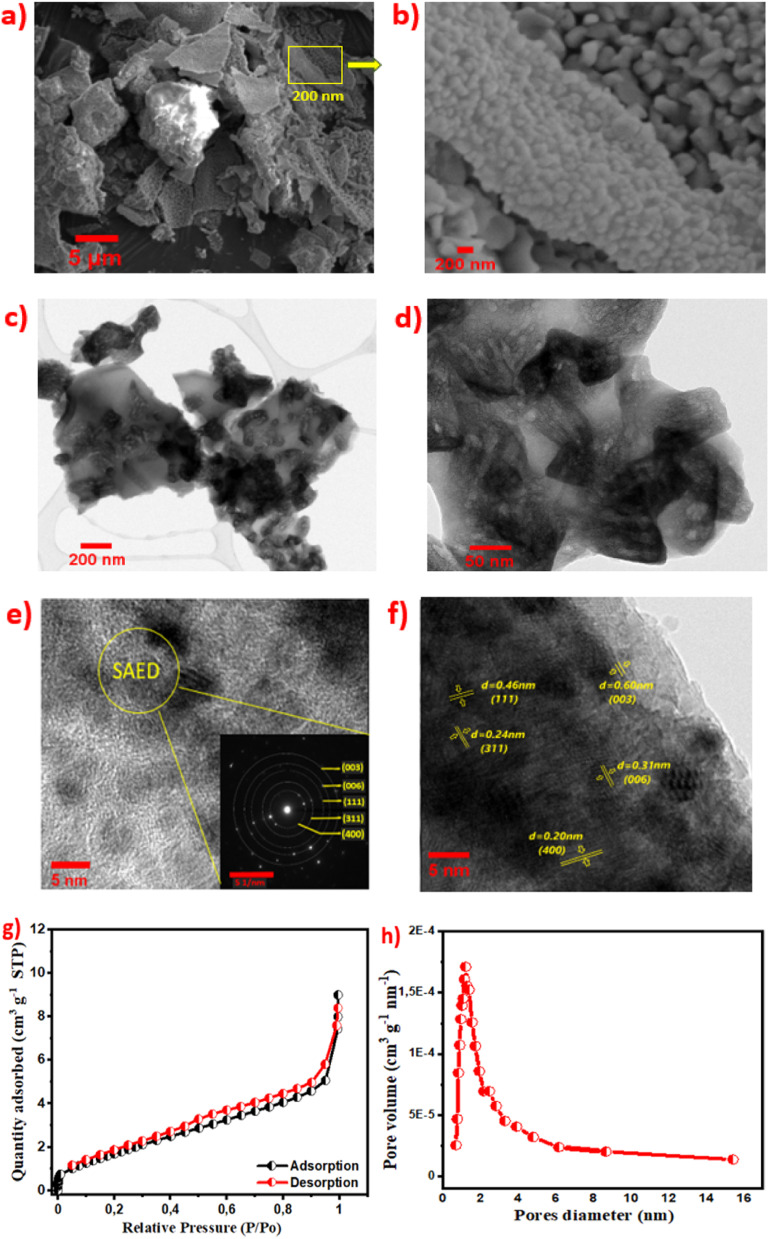
(a and b) SEM and (c–f) TEM (with SAED) images, (g) absorption/desorption N_2_ isotherm, (h) pore size distribution of the NiCoO@BC(4 h) composite.

The morphological features from SEM images (low and high magnifications) of the NiCoO@BC(4 h) composite display microsheet-like structures, as shown in Fig. 4a and b. The micrographs exhibit nanograins (20–50 nm) grown on random sheets. Energy dispersive X-ray spectroscopy (EDS) is used to obtain further information about the distribution of chemical elements within the composite. The mapping images represented in Fig. S5† show that carbon, cobalt, nickel, nitrogen, and oxygen elements are homogeneously distributed in the NiCoO@BC(4 h) composite.

For further morphological analysis, transmission electron microscopy (TEM) was performed. Nanograins and a transparent zone referring to the carbon material (Fig. 4c and d)^72^ are clearly observed. The enlarged high-resolution images in Fig. 4e and f exhibit the presence of defined fringes in the composite structure. The distances between fringes (0.20, 0.24, and 0.46 nm) correspond to the (400), (311), and (111) planes of NiCo_2_O_4_ while those at 0.31 nm and 0.60 nm are associated with the (006) and (003) planes of the NiOOH hexagonal structure, in agreement with the XRD results.^73,74^ Well-defined diffraction rings are also displayed by SAED (selected area electron diffraction) inserted in Fig. 4e, indicating that the nanomaterials possess a polycrystalline structure.^75^

The distribution of the NiCoO@BC(4 h) pore size was examined with N_2_ adsorption/desorption analysis. Fig. 4g displays an isotherm type IV, with hysteresis loops of H4 according to the IUPAC classification.^17,45,76^ NiCoO@BC(4 h) displays a specific surface area of 5.2 m^2^ g^−1^ from BET analysis.^77,78^ The corresponding pore size distribution shown in Fig. 4h indicates that most pores lie in the micro and mesopores range. This mesoporous structure enhances the diffusion of electrolyte ions, which improves charge transport and provides more electroactive sites. This feature is advantageous for energy storage.^28,60^

### 5.2. Electrochemical studies

Electrochemical measurements of NiCoO and NiCoO@BC composites were carried out by cyclic voltammetry (CV), galvanostatic charge/discharge (GCD), and electrochemical impedance spectroscopy (EIS) using aqueous potassium hydroxide KOH (6 M) electrolyte.

The choice of this electrolyte was guided by its excellent properties for electrochemical studies, as shown in Table 1. KOH stands out for its high ionic conductivity (73.5 cm^2^ Ω mol^−1^ for K^+^) and small hydration sphere radius compared to neutral and acidic electrolytes (LiCl, KNO_3_, Na_2_SO_4_, and KCl).^79–82^ KOH concentration was also optimised by Xueqin Lang *et al.*^83^ using 1 M, 3 M, and 6 M KOH for La_0.85_Sr_0.15_MnO_3_@NiCo_2_O_4_ composite. They concluded that 6 M KOH gave better electrochemical performances. At a current density of 32 A g^−1^, the material provided an optimal capacitance of 1341 F g^−1^ (with capacitance retention of 52%) in 6 M KOH electrolyte compared to 760 F g^−1^ (38% of capacitance retention) in 3 M and 400 F g^−1^ (10% of capacitance retention) in 1 M KOH.^82^

**Table 1:** Radius of the hydration sphere, molar conductivity, and ionic mobility of different ions

Item	Ion			
	OH^−^	Cl^−^	NO_3_^−^	SO_4_^2−^
Radius of hydration sphere (Å)	3.00	3.32	3.35	3.79
Molar conductivity (cm^2^ Ω mol^−1^)	198.00	76.34	71.44	79.80
Ionic mobility (*μ* × 10^−5^ cm^2^ s^−1^ V^−1^)	20.60	7.91	4.40	8.30

**Fig. 5:**
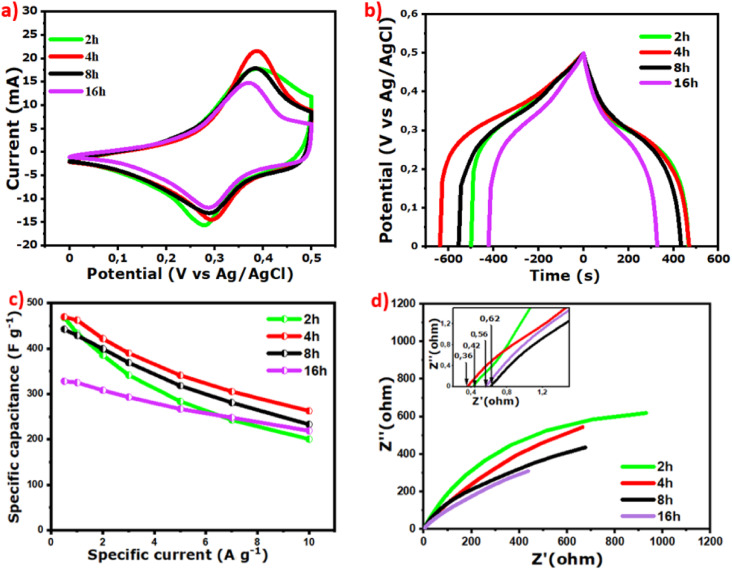
(a) CV plots at 5 mV s^−1^, (b) GCD plots at 0.5 A g^−1^, (c) *C*_s_*versus* current density, and (d) EIS of NiCoO@BC samples for 2 h, 4 h, 8 h, and 16 h synthesis time durations.

The working potential of all the samples was first optimized to be 0.5 V using the reference electrode (Ag/AgCl). Fig. 5a compares CV plots of the composites at different synthesis time durations (2 h, 4 h, 8 h, 16 h). The CV of the materials performed at a rate of 5 mV s^−1^ exhibits high current responses and faradaic behavior with cathodic oxidation and anodic reduction peaks.^61^ The large curve areas also imply a capacitance contribution of the carbon-based material combined with reversible redox reactions of the metal oxides involving redox couples Co^3+^/Co^2+^ and Ni^3+^/Ni^2+^ into the composites, as suggested by XPS results. Previous studies have described the redox reaction mechanism using the following eqn (1) and (2).^19,84^

1 NiCo_2_O_4_ + H_2_O + OH^−^ ↔ NiOOH + 2CoOOH + e^−^

2 NiOOH + OH^−^ ↔ NiO_2_ + H_2_O + e^−^

Fig. 5b presents the GCD of all the samples recorded at 0.5 A g^−1^ over the same potential window of 0.5 V. The nonlinear curves confirm the faradaic behavior of the composites. GCD curves also display symmetric shapes, indicating the reversibility of the redox reactions during charging–discharging. Fig. 5c presents the specific capacitances *versus* current densities calculated from GCD using eqn (3):3
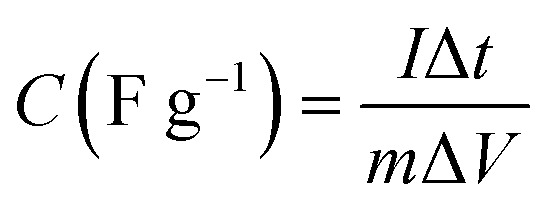
*I* (A) is the galvanometric current, *C* (F g^−1^) is the specific capacitance, Δ*V* (V) is the working potential, Δ*t* (s) is the discharge time, and *m* (g) is the mass of the active material on the electrode.

The electrode mass loading of the samples for 2 h, 4 h, 8 h, and 16 h synthesis time durations are determined to be around 2.7 mg (∼2.7 mg cm^−2^), and the specific capacitances at 0.5 A g^−1^ are calculated to be 468, 475, 442, and 328 F g^−1^, respectively. In addition, 43%, 56%, 53%, and 66% of their value are retained from 0.5 to 10 A g^−1^ of current density.

Electrochemical impedance spectroscopy was further performed to provide a more comprehensive understanding of the material properties. EIS plots of the composites in Fig. 5d display a steep slope at low frequencies, revealing the existence of Warburg impedance (*W*) from electrolyte species diffusion into the electrode material.^61,85^ The inserted Nyquist plots show that all the composite electrodes possess low resistance. The analyzed curves exhibit ESRs of 0.42, 0.36, 0.62, and 0.56 ohm for the samples at 2 h, 4 h, 8 h and 16 h, respectively. These low ESR values prove the importance of the biomass carbon-based material in the composites. In particular, it can be noted that the NiCoO@BC(4 h) sample with more carbon content (as shown in Fig. 3a) exhibits lower ESR and better current response, specific capacitance, and capacitance retention than samples with other time durations (2 h, 8 h, 16 h).

Fig. S6† compares the electrochemical performances of the NiCoO@BC(4 h) composite with those of pristine NiCoO. As shown in Fig. S6a,† the composite displays a superior current response compared to the pristine sample. The specific capacitances of the pristine sample and NiCoO@BC(4 h) composite from GCD (Fig. S6b†) are presented as a function of the current density in Fig. S6c.† The NiCoO@BC(4 h) composite retains more than 56% of its capacitance from 0.5 to 10 A g^−1^, while the pristine sample suffers from a sharp capacitance drop, losing almost 80% of its capacitance value even at 2 A g^−1^. The NiCoO@BC(4 h) composite also displays a lower ESR (0.36 ohm) than the pristine sample (1.00 ohm) (Fig. S6d†), which is efficient to enhance the charge transfer and improve the capacitance.^68^ The good capacitance, conductivity, and rate capability can be assigned to the effective incorporation of nitrogen and carbon from biomass into the metal oxide matrix.

**Fig. 6:**
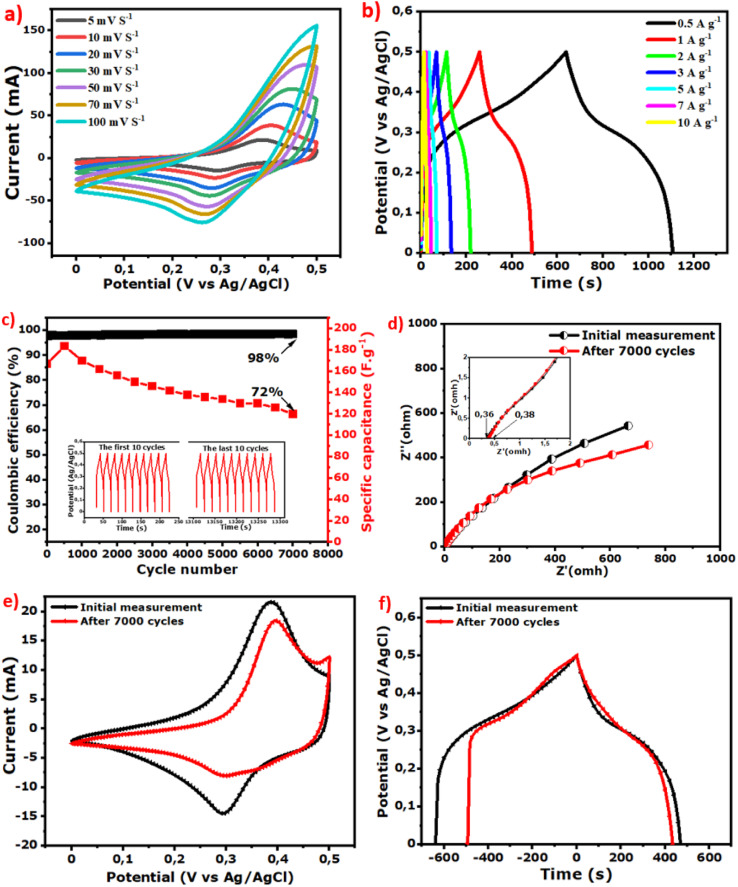
(a) CV curves from 5 mV s^−1^ to 100 mV s^−1^, (b) GCD profiles from 0.5 A g^−1^ to 10 A g^−1^, (c) capacitance retention and coulombic efficiency after 7000 cycles at 10 A g^−1^ (d) EIS, (e) CV curves at 5 mV s^−1^ and (f) GCD profiles at 0.5 A g^−1^ after 7000 cycles compared to the initial measurements of the NiCoO@BC(4 h) composite.

The CV curves of NiCoO@BC(4 h) obtained at various scans from 5 mV s^−1^ to 100 mV s^−1^ (see in Fig. 6a) show the existence of redox peaks. These oxidation and reduction peaks located at around 0.41 V and 0.28 V barely shift as the scan rate increases. However, their shape remains similar, suggesting that the electrode performs well at high scan rates. The GCD profiles between 0.5 A g^−1^ and 10 A g^−1^ are nonlinear, which confirms the faradaic behavior of the composite.^86^

Table 2 compares the results of the present work with some results reported in the literature. The obtained composites synthesized with the facile energy-saving process using biomass carbon display a good specific capacitance as compared to these other works. The stability was also tested by continuous GCD at 10 A g^−1^ up to 7000 cycles, and the corresponding stability curves are represented in Fig. 6c. It is observed that the NiCoO@BC(4 h) sample shows 98% of coulombic efficiency and maintains 72% of its initial capacitance even after 7000 cycles, which indicates a good cycle life. The first 10 cycles, compared to the last 10 cycles of charging–discharging inserted in Fig. 6c, show no difference in cycle shape at a stable voltage of 0.5 V, confirming the excellent material stability. EIS, CV, and GCD measurements were performed after 7000 cycles and compared with the initial measurements. The ESR shown in Fig. 6d remains similar (0.38 Ω *vs.* 0.36 Ω) with a slow increase of diffusion resistance exhibited by the slight curve shift towards the *X* axis at low frequency. The CV curves show that the faradaic behavior of the composite is maintained with a slight decrease of the redox peaks, as shown in Fig. 6e. The GCD profile in Fig. 6f displays practically the same shape after 7000 cycles, indicating that the electrode performances are almost conserved.

**Table 2:** Summary of electrochemical performances in comparison with some reported works

Electrodes	KOH electrolytes	Specific capacitances	Capacitance retention	Cycle number	References
MnCo_2_O_4.5_	2 M	321.0 F g^−1^	87.0%	4000	19
NiCo_2_O_4_	1 M	294.0 F g^−1^	89.8%	3000	28
MnCo_2_O_4_/carbon	3 M	235.6 F g^−1^	86.0%	5000	87
NiCo_2_O_4_@g-C_3_N_4_(C)	6 M	325.7 F g^−1^	93.6%	2000	39
Pristine NiCoO	6 M	353.0 F g^−1^	51.0%	4000	This work
NiCoO@BC(4 h)	6 M	475.0 F g^−1^	86.0%	4000	This work
			72.0%	7000	

To further examine the practicality of the NiCoO@BC composite, an ASC (NiCoO@BC(4 h)//AC) was assembled, with AC as the negative electrode and NiCoO@BC(4 h) composite as the positive electrode. The electrochemical performances of AC were tested in a three-electrode system, as shown in Fig. S7.† At 5 mV s^−1^, the CV plots in the potential window from −1 to 0.2 V exhibit an ideal capacitive behavior indicated by rectangular-like profiles (Fig. S7a†).^88^ The linear GCD curves at different current density values from 1 to 10 A g^−1^ correspond to a double-layer capacitive charge storage (Fig. S7b†).^13^ AC exhibits a specific capacitance of 160 F g^−1^ at 0.5 A g^−1^ and retains it well at a high current density of 10 A g^−1^ (Fig. S7c†) while the specific capacitance increases by 18% after 7000 cycles (Fig. S7d†).

**Fig. 7:**
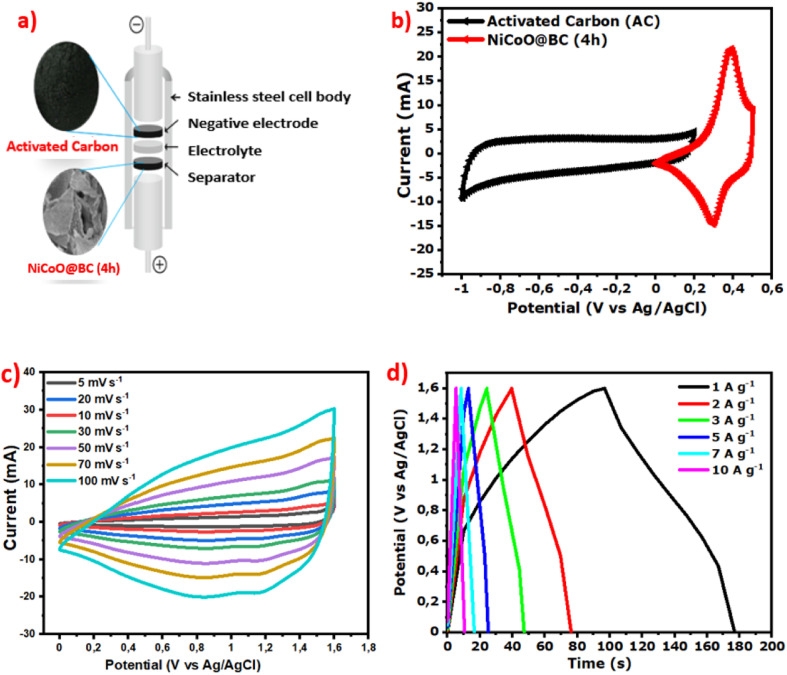
(a) Assembled full device, (b) CV plots of NiCoO@BC(4 h) and AC, (c) CV plots from 5 to 100 mV s^−1^, and (d) GCD profiles from 1 to 10 A g^−1^ of the device.

The assembled full device using a Swagelok system (stainless steel plungers) and filter paper as the separator is schematically represented in Fig. 7a, and the CV of both composite and AC are displayed in Fig. 7b. The mass ratio of NiCoO@BC(4 h) (3.6 mg) and AC (4.4 mg) was calculated to be 0.8 based on the charge balance using eqn (4).4
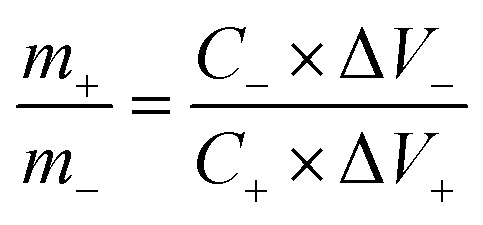
*m* (g) is the mass of electrode materials, *C* (F g^−1^) is the specific capacitance, and Δ*V* (V) is the potential window.

Fig. 7c exhibits the CV curves obtained with scan rates varying from 5 to 100 mV s^−1^ at a stable voltage of 1.6 V. The curves present a good current response and a large area indicating both faradaic and EDLC contributions corresponding to a hybrid-type supercapacitor. The nonlinear GCD plots in Fig. 7d at different current densities illustrate the faradaic contribution by the composite material, confirming the hybrid behavior of the device.

**Fig. 8:**
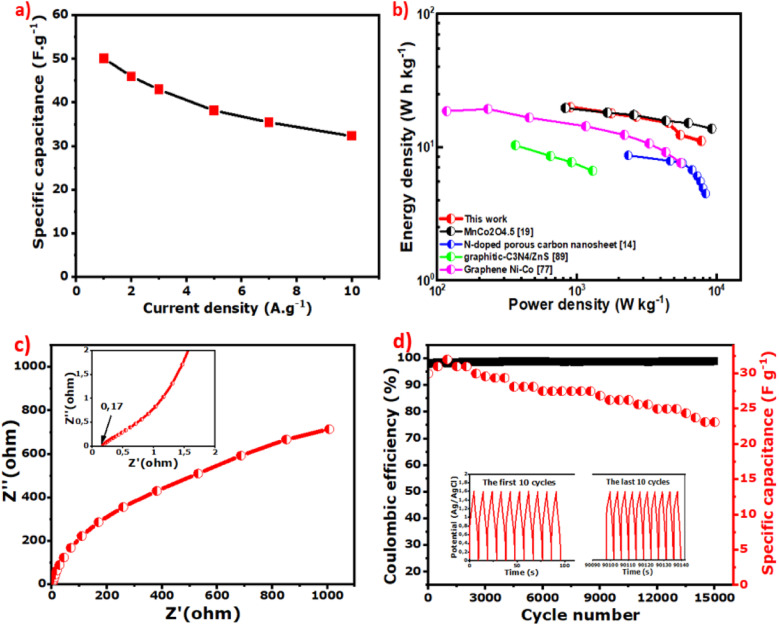
(a) Specific capacitance *versus* current density, (b) Ragone plot of *E*_d_ as a function of *P*_d_, (c) EIS curves (the inset is the ESR), and (d) capacitance retention and coulombic efficiency of the ASC device (NiCoO@BC(4 h)//AC) for up to 15 000 cycles.

The specific capacitance of the ASC calculated at 1 A g^−1^ from GCD is 50.2 F g^−1^. When plotted *versus* current density, 65% of this value remains even at 10 A g^−1^, which corresponds to a good rate capability (Fig. 8a). From GCD, the energy density (*E*_d_) values and the corresponding power density (*P*_d_) are also calculated using eqn (5) and (6) and plotted in a Ragone plot (Fig. 8b).5
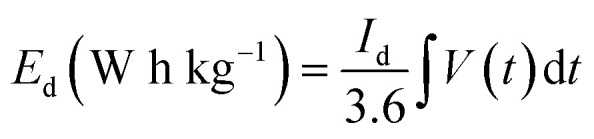
6
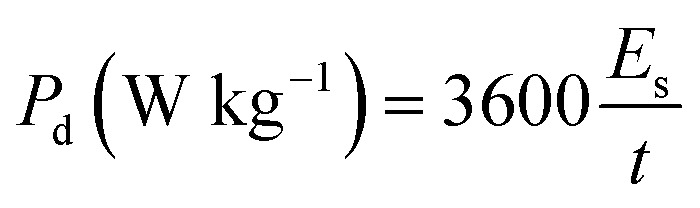
*V* (V) is the potential window, *t* (s) is the discharge time, and *I*_d_ (A g^−1^) is the current density.

The *E*_d_ and *P*_d_ of the ASC calculated at 1 A g^−1^ are 20 W h kg^−1^ and 900 W kg^−1^, respectively, and are superior or at least comparable to values reported in the literature, as shown in Fig. 8b.^14,19,77,89,90^

The EIS results of the device in Fig. 8c exhibit a low ESR of 0.17 Ω due to the synergy between the EDLC properties of AC for the negative electrode and the NiCoO@BC composite as a faradaic material for the positive electrode. The life cycle of the ASC, a crucial parameter for practical application, was evaluated at 10 A g^−1^ with 15 000 cycles (Fig. 8d). During the first 900 cycles, an increase in capacitance is observed. This could be because the electrode is gradually activated by the diffusion of electrolyte species within the material. The capacitance decreases slightly in the subsequent cycles because the whole device is fully activated and reaches its maximum wettability at 900 cycles, as explained by Q. Li *et al.*^17^ The NiCoO@BC(4 h)//AC device shows an excellent coulombic efficiency of 99%, capacitance retention of 78% even after 15 000 cycles at 10 A g^−1^, and no difference in cycle shape from the first to the last 10 charge–discharge cycles at a potential of 1.6 V, as shown in the inset of Fig. 8d.

## 6. Conclusion

A green hydrothermal process was successfully developed to combine biomass carbon from the bark of AO with nickel cobaltite hydroxide NiCo_2_O_4_/NiOOH (NiCoO@BC) at low temperatures for different synthesis time durations. The highest amount of carbon was observed with the sample synthesized in 4 h. The synergy between metal oxide nanoparticles and biomass carbon allows good electrochemical performances. The NiCoO@BC(4 h) composite displays a low ESR of 0.36 Ω, good specific capacitance of 475 F g^−1^ at 0.5 A g^−1^, as well as good stability. NiCoO@BC(4 h) was tested as a positive electrode in a full device with AC as a negative electrode. The system exhibits promising electrochemical performances for practical applications: a large potential window of 1.6 V, an energy density of 20 W h kg^−1^, and a power density of 900 W kg^−1^. The device also revealed good stability by retaining 99% coulombic efficiency, 65% of its specific capacitance from 0.5 A g^−1^ to 10 A g^−1^, and a capacitance retention of 78% even after 15 000 cycles. Therefore, these promising results of the composite of nickel–cobalt oxide (NiCoO) and biocarbon derived from the bark of AO for supercapacitor electrodes make this technology suitable for clean energy for several applications, such as electronics, hybrid electric vehicles, *etc.*

## Conflicts of interest

The authors declare that they have no known competing financial interests or personal relationships that could have appeared to influence the work reported in this paper.

## Supplementary Material

RA-014-D3RA08138A-s001
