# Supplementary material for: High electrochemical performance of nickel cobaltite@biomass carbon composite (NiCoO@BC) derived from the bark of Anacardium occidentale for supercapacitor application
Source: RSC Adv. 2024 Feb 14;14(9):5782–96. doi: 10.1039/d3ra08138a (PMC10865185; doi:10.1039/d3ra08138a)
Supplement: RA-014-D3RA08138A-s001 [file RA-014-D3RA08138A-s001.pdf]

Electronic Supplementary Material (ESI) for RSC Advances.
This journal is © The Royal Society of Chemistry 2024

Electronic Supplementary Information (ESI)

High electrochemical performance of the nickel cobaltite@biomass carbon composite (NiCoO@BC) derived from bark of Anacardium Occidentale for supercapacitor application

Modou Diop[1,2], Baye Modou Ndiaye[1,2], Sokhna Dieng[2], Balla D. Ngom[2*], Mohamed Chaker[1*]

[1] Institut National de la Recherche Scientifique (INRS), Centre – Énergie Matériaux Télécommunications (EMT) 1650, Boul. Lionel Boulet, Varennes (Québec) J3X 1P7 Canada

[2] Laboratoire de Photonique Quantique, d'Énergie et de Nano-Fabrication (LPQEN), Faculté des Sciences et Techniques Université Cheikh Anta Diop de Dakar (UCAD) B.P. 5005 Dakar-Fann Dakar, Sénégal

*Corresponding authors.
Email: bdngom@gmail.com
Email: mohamed.chaker@inrs.ca

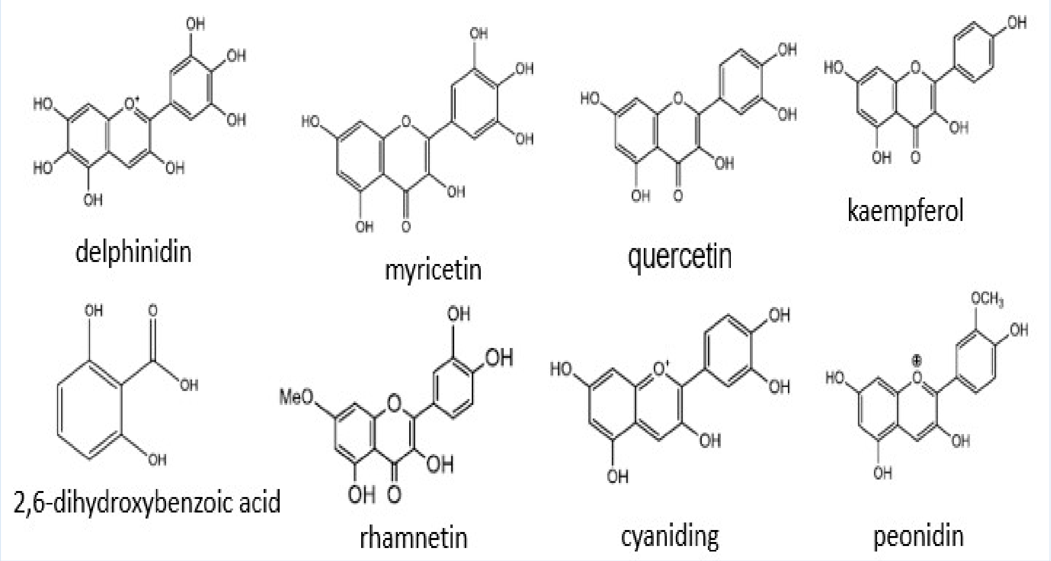

Fig. S1: Some of the chemical bioactive molecular compounds (phenolic acid) within the Anacardium Occidentale (AO).

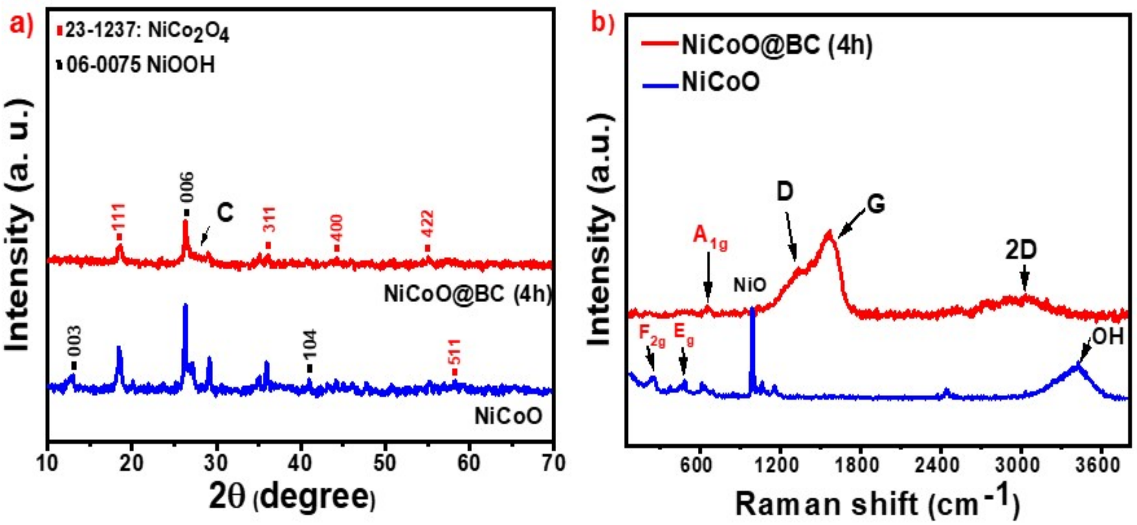

Fig. S2: XRD (a) and Raman spectra (b) of pristine NiCoO and NiCoO@BC (4h) composite.

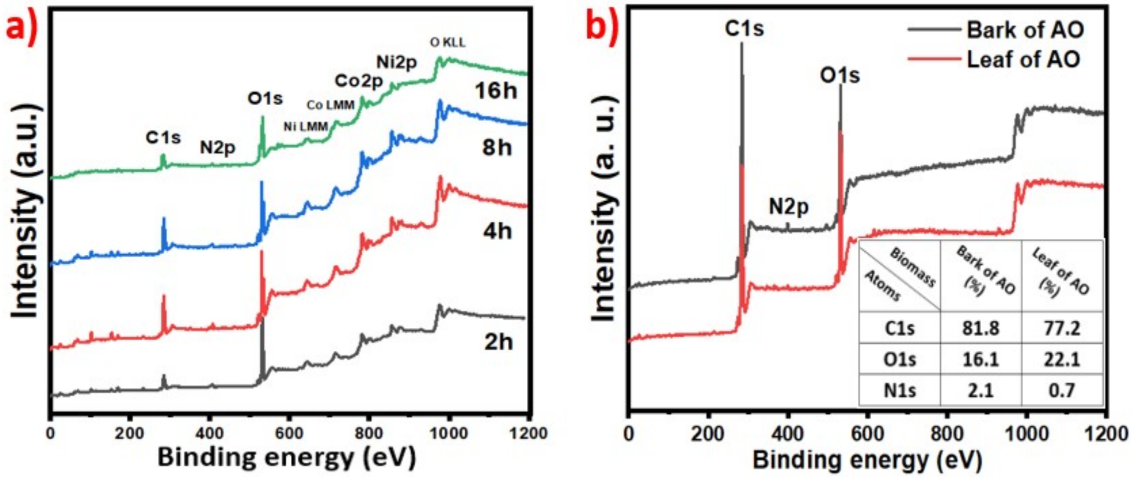

Fig. S3: a) XPS survey spectra of the NiCoO@BC composites at different synthesis time durations (2h, 4h, 8h, 16h) and b) of the leaf and the bark of AO.

Table S1: Chemical composition of the leaves and barks of AO in comparison with some parts of other biomass.

**Sample**	**C (%)**	**H (%)**	**N (%)**	**O (%)**	**References**
Raw Willow	49.5	6.1	0.2	44.4	Raimie H.H. Ibrahim et al.
Raw eucalyptus	49.3	6.5	0.0	44.3	
Raw hardwood	46.8	5.9	0.1	47.2	
Raw softwood	46.7	5.9	0.0	47.4	
Bark of AO	81.8	-	2,1	16,1	This work
Leaf of AO	77,2	-	0,7	22,1	

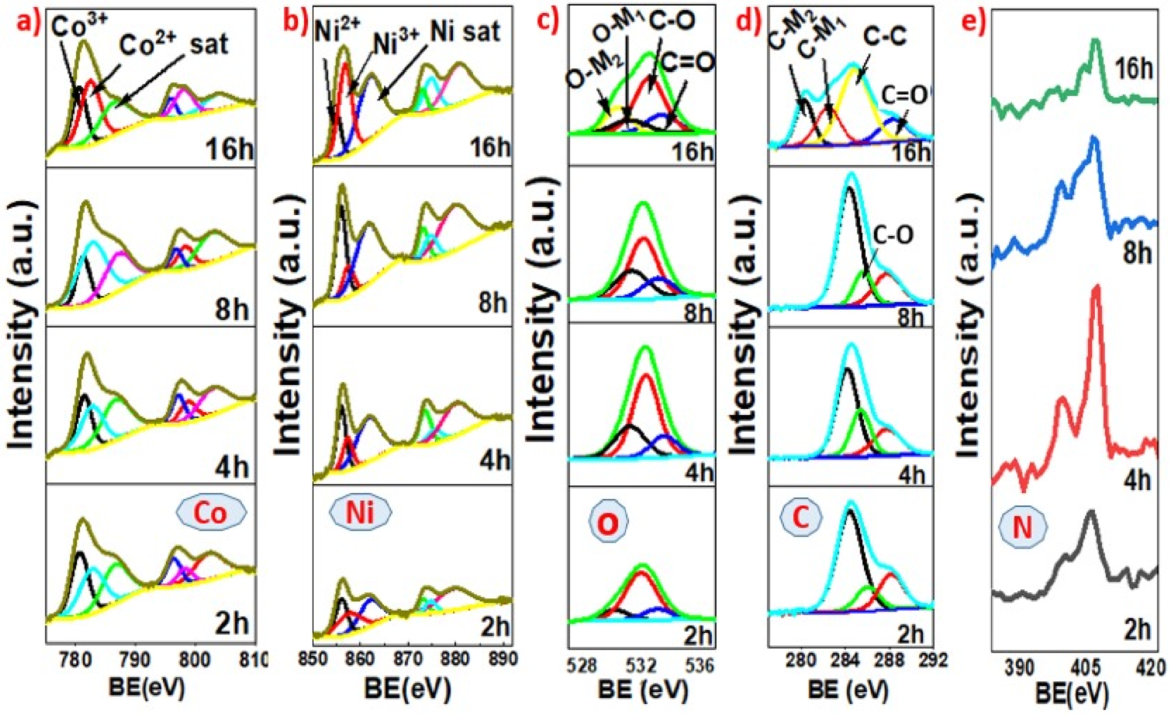

Fig. S4: High resolution XPS spectra of elements (a) Co, (b) Ni, (c) O, (d) C and (e) a zoom on N peak of the NiCoO@BC composites at different synthesis time durations (2h, 4h, 8h, 16h).

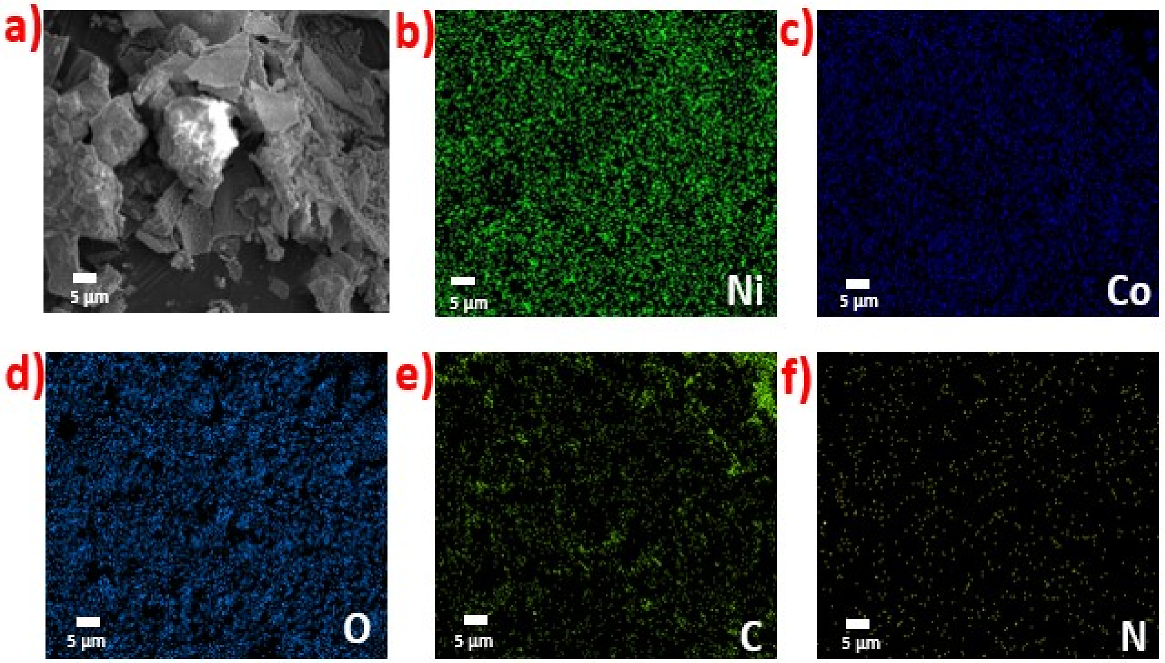

Fig. S5: (a) SEM and mapping images of the element distribution of (b) Ni, (c) Co, (d) O, (e) C and (e) N of the NiCoO@BC(4h) composite.

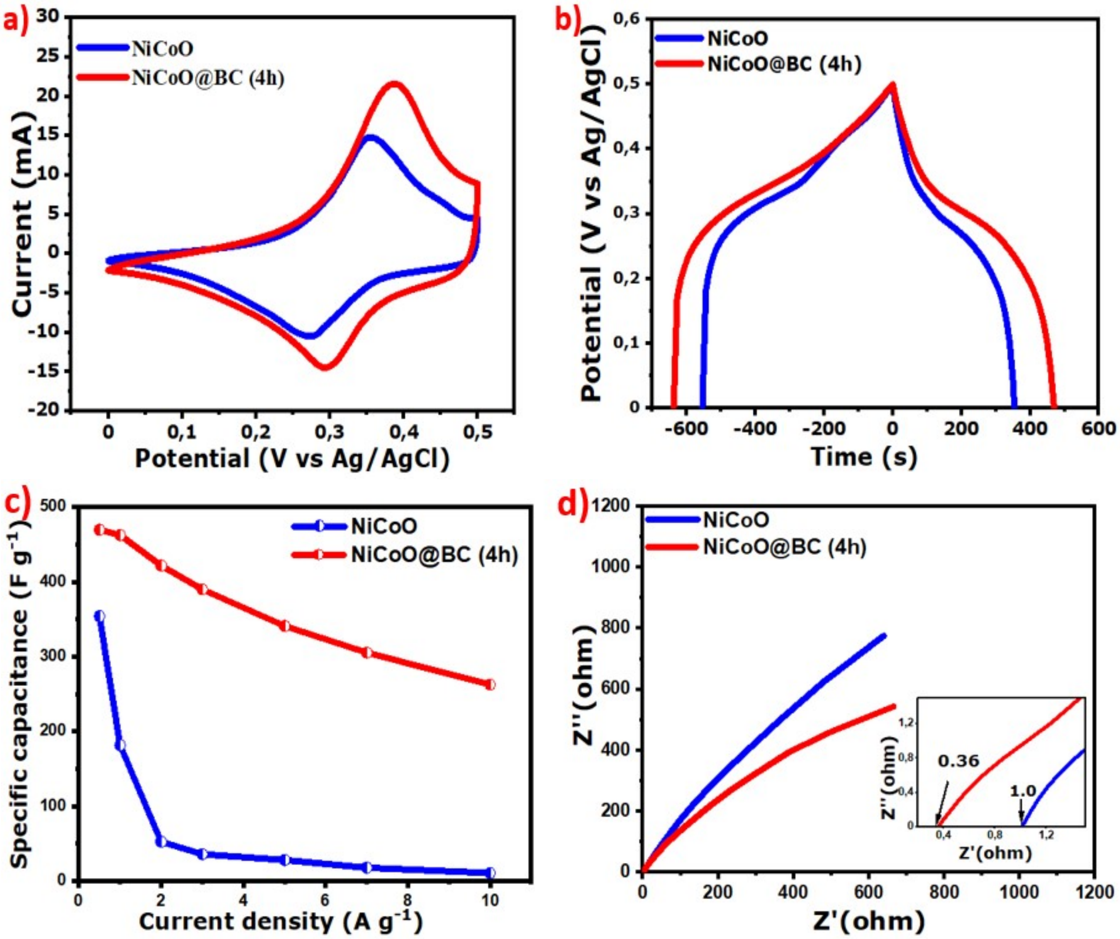

Figure S6: (a) CV curves at 5 mV s^{-1}, (b) GCD profiles at current density of 0.5 A g^{-1}, (c) specific capacitance versus current density and (d) EIS data for NiCoO@BC(4h) composite and pristine NiCoO.

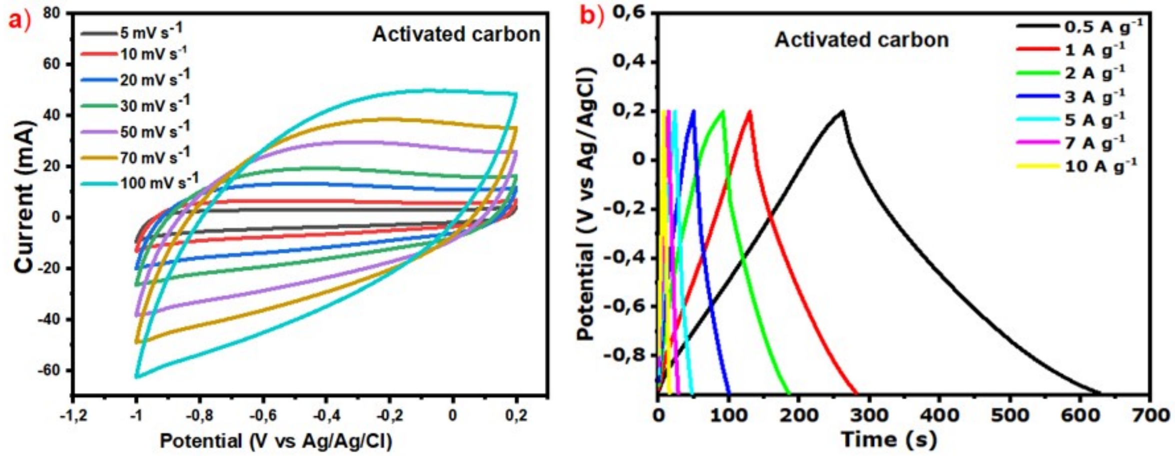

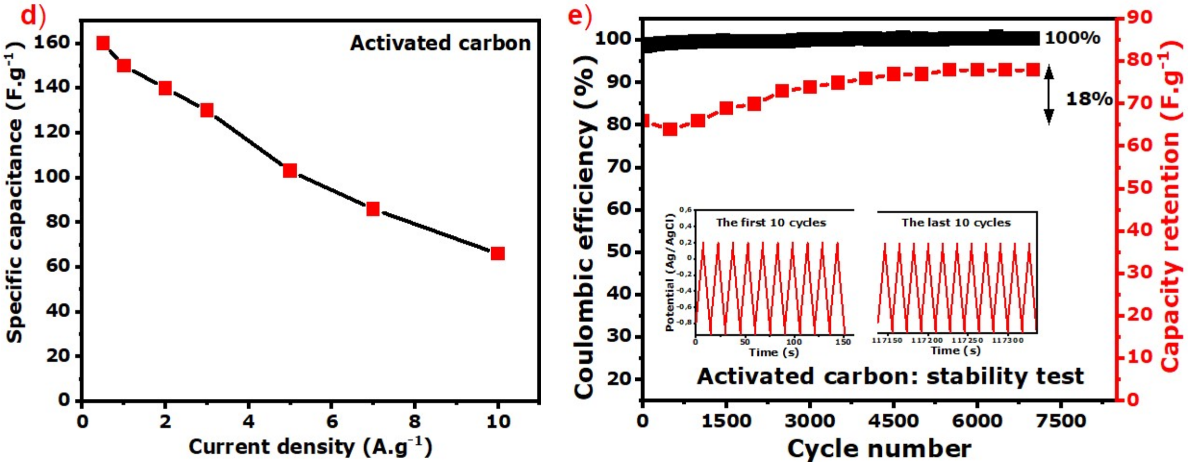

Figure S7: (a) CV, (b) GCD, (c) specific capacitance versus current density and (d) stability test of the Activated Carbon (AC).
